# Whole grain intake associated molecule 5-aminovaleric acid betaine decreases β-oxidation of fatty acids in mouse cardiomyocytes

**DOI:** 10.1038/s41598-018-31484-5

**Published:** 2018-08-29

**Authors:** Olli Kärkkäinen, Tomi Tuomainen, Ville Koistinen, Marjo Tuomainen, Jukka Leppänen, Tuomo Laitinen, Marko Lehtonen, Jaana Rysä, Seppo Auriola, Antti Poso, Pasi Tavi, Kati Hanhineva

**Affiliations:** 1 Institute of Public Health and Clinical Nutrition, University of Eastern Finland, P.O. Box 1627, FI-70211 Kuopio, Finland; 2 A.I.Virtanen Institute for Molecular Sciences, University of Eastern Finland, P.O. Box 1627, FI-70211 Kuopio, Finland; 3 School of Pharmacy, University of Eastern Finland, P.O. Box 1627, FI-70211 Kuopio, Finland; 4 Department of Internal Medicine VIII, University Hospital Tübingen, Otfried-Müller-Strasse 10, 72076 Tübingen, Germany; 5 Pharmazeutische Chemie, Pharmazeutisches Institut, Eberhard-Karls-Universität Tübingen, Auf der Morgenstelle 8, D-72076 Tübingen, Germany

## Abstract

Despite epidemiological evidence showing that diets rich in whole grains reduce the risk of chronic life-style related diseases, biological mechanisms for these positive effects are mostly unknown. Increased 5-aminovaleric acid betaine (5-AVAB) levels in plasma and metabolically active tissues such as heart have been associated with consumption of diets rich in whole grains. However, biological effects of 5-AVAB are poorly understood. We evaluated 5-AVAB concentrations in human and mouse heart tissue (3–22 µM and 38–78 µM, respectively) using mass spectrometry. We show that 5-AVAB, at physiological concentration range, dose-dependently inhibits oxygen consumption due to β-oxidation of fatty acids, but does not otherwise compromise mitochondrial respiration, as measured with oxygen consumption rate in cultured mouse primary cardiomyocytes. We also demonstrate that this effect is caused by 5-AVAB induced reduction of cellular L-carnitine. Reduced L-carnitine levels are at least partly mediated by the inhibition of cell membrane carnitine transporter (OCTN2) as evaluated by *in silico* docking, and by siRNA mediated silencing of OCTN2 in cultured cardiomyocytes. 5-AVAB caused inhibition of β-oxidation of fatty acids is a novel mechanism on how diets rich in whole grains may regulate energy metabolism in the body. Elucidating potentially beneficial effects of 5-AVAB *e.g*. on cardiac physiology will require further *in vivo* investigations.

## Introduction

Epidemiological evidence shows that diets rich in whole grains reduce the risk of chronic life-style related diseases, including cardiovascular disorders^1–4^. Despite the wealth of evidence in population level, the biological mechanisms responsible for the protective effect of whole grains are mostly unknown. A recent investigation identified a group of trimethylated compounds that were associated with consumption of diets rich in whole grains^5^. One of these compounds, 5-aminovaleric acid betaine (5-AVAB, 5-trimethylaminovaleric acid), accumulated in metabolically active tissues in mice including heart, muscle and brown adipose tissue. However, 5-AVAB is rarely reported from any mammal-based studies, and thus there is only limited information available about its biological functions.

5-AVAB has similar molecular structure with meldonium (mildronate), a drug that inhibits β-oxidation of fatty acids and has been associated with improved cardiac mitochondrial function after ischemia^6^. β-oxidation is a mitochondrial process by which co-factors for citric acid cycle and electron transport chain are generated from fatty acids. Meldonium reduces cellular L-carnitine levels, which is needed in β-oxidation, by blocking cell membrane carnitine transporter (OCTN2) and by reducing biosynthesis of L-carnitine from γ-butyrobetaine by inhibiting γ-butyrobetaine hydroxylase (BBOX)^6,7^. In a previous study, where several structural analogues of L-carnitine were tested in CCL 27 heart cell lines, 5-AVAB (named 5-trimethylaminovaleric acid in the report) reduced L-[Me-^3^H]carnitine intake^8^. Moreover, in a study where structural analogues of meldonium were analysed, 5-AVAB (named 5-[trimethylammonio]pentanoate in the report) did not seem likely to inhibit biosynthesis of L-carnitine via BBOX^7^.

Our hypothesis was that 5-AVAB, like meldonium, reduces β-oxidation of fatty acid by blocking OCTN2. First, we measured 5-AVAB concentrations in human and mouse heart tissue to evaluate an estimate of physiological concentration range. Then we investigated the effects of these physiological concentrations of 5-AVAB on energy metabolism in primary mouse cardiomyocytes. Furthermore, we established an *in silico* model to evaluate if OCTN2 is a possible molecular target of 5-AVAB and further verified this by silencing OCTN2 in cardiomyocytes cultures.

## Results and Discussion

### Physiological concentrations of 5-AVAB reduce β-oxidation of fatty acids

In order to define physiological concentrations of 5-AVAB, we examined human and mouse heart tissue with the targeted mass spectrometry method. In the human heart samples, 5-AVAB concentrations were 15 µmol/g, 18 µmol/g and 22 µmol/g in the ventricular samples, and 3 µM and 8 µM in the atrial samples. In the mouse whole heart samples, 5-AVAB concentrations ranged from 38 µmol/g to 78 µmol/g (mean = 55 µmol/g, SD = 19 µmol/g).

**Figure 1:**
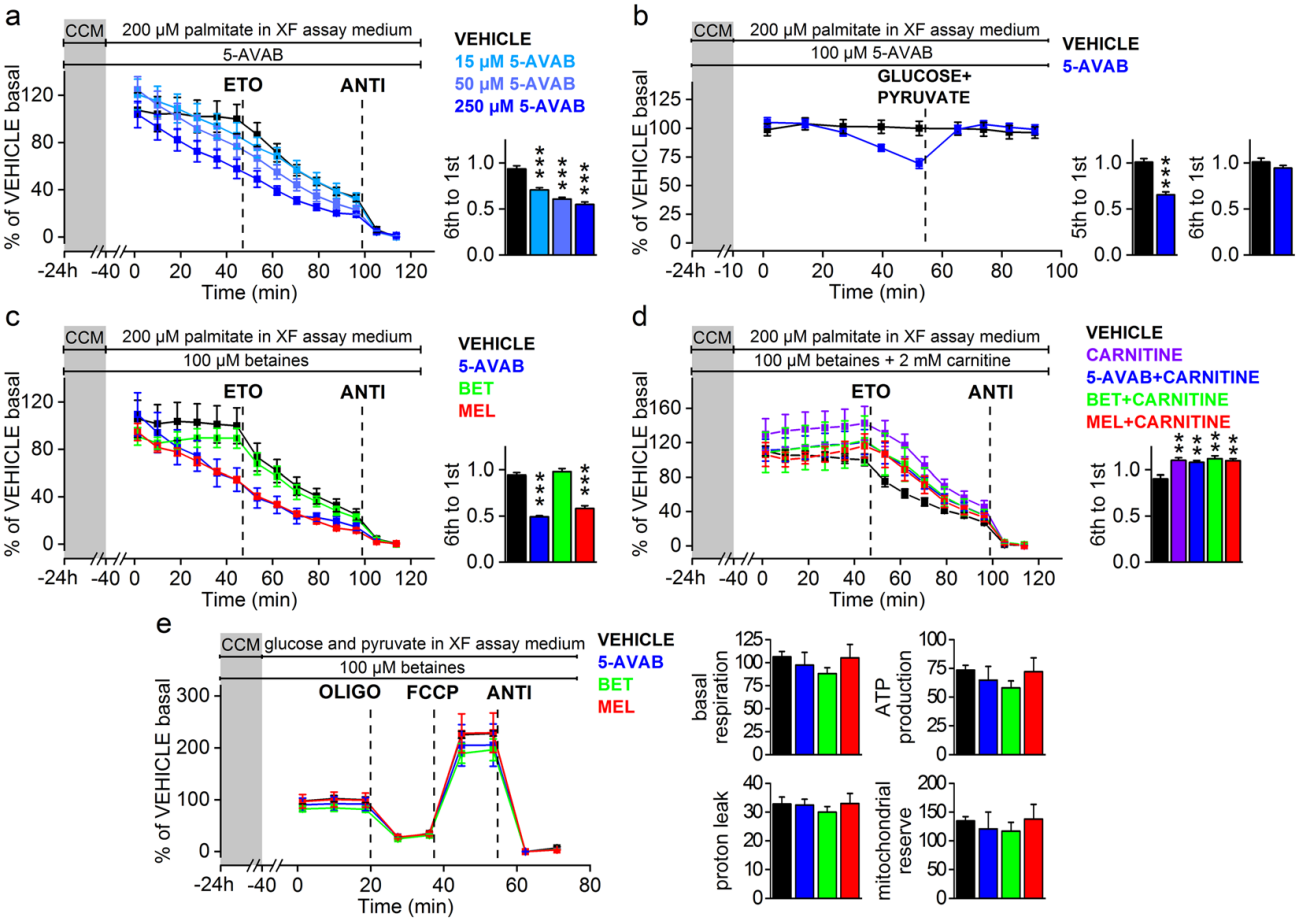
5-AVAB inhibits β-oxidation in cultured mouse cardiomyocytes. Effect of 5-AVAB treatment to mitochondrial respiration was assessed in cardiomyocytes. (**a**–**d**) After 24 h treatment in cardiomyocyte culture medium, which contains glucose as energy substrate, solution was changed to assay medium with palmitic acid as an energy source. (**a**) Treatment with different concentrations of 5-AVAB shows dose-dependent effect on palmitic acid oxidation as assessed by ratio of 6th and 1st measurement in individual assay wells (mean ± SEM). (**b**) This effect can be reversed by addition of glucose and pyruvate. (**c**) 5-AVAB has similar effect on the use of palmitic acid as meldonium, whereas glycine betaine shows no effect. (**d**) Addition of carnitine to the medium blocks the effect of 5-AVAB and meldonium. (**e**) In the mitochondrial stress test, none of the compounds had effect on the parameters of mitochondrial respiration in presence of glucose and pyruvate. CCM, cardiomyocyte culture medium; ETO, etomoxir; ANTI, antimycin A; MEL, meldonium; BET; glycine betaine; OLIGO, oligomycin; FCCP, carbonyl cyanide-4-trifluoromethoxy-phenylhydrazone. **p < 0.01; ***p < 0.001.

Next, we measured the effect of 5-AVAB on the β-oxidation of cultured cardiomyocytes with Seahorse extracellular flux analyser. We noticed that when cells exposed to 5-AVAB for 24 h were supplemented with medium containing palmitate as a sole energy source prior to the Seahorse assay, the oxygen consumption rate (OCR) decreased during the assay, whereas in vehicle treated cells OCR remained constant (Fig. 1). Observed decrease in the OCR is probably a result of decreasing cellular glucose storage as the cells treated with 5-AVAB have impaired capability to employ fatty acids from the assay medium in the mitochondrial respiration. Therefore, we used the ratio of OCR in sixth measurement (time point 84 min after palmitate supplementation) to OCR in first measurement (41 min after palmitate supplementation) as a measure of cells’ ability to utilize mitochondrial β-oxidation. After determining palmitate fuelled OCR, carnitine palmitoyltranferase inhibitor etomoxir was injected to cells to confirm that the observed respiration originated from fatty acid oxidation. Finally, antimycin A was injected to cells to completely block mitochondrial respiration. Minimum OCR after antimycin A injection was subtracted from each measured OCR value to remove the non-mitochondrial respiration from the results.

5-AVAB caused a significant dose-dependent reduction in oxygen consumption, indicating a reduced use of fatty acids in mitochondrial ATP production (Fig. 1a). After the addition of etomoxir, similar decrease in the respiration of vehicle treated cells was observed as in the first six measurements in the 5-AVAB treated cells. Since etomoxir acutely causes situation comparable to L-carnitine deficiency, this further suggests that 5-AVAB induced reduction of L-carnitine is indeed causing the response. Addition of glucose and pyruvate rescued the cell respiration in a similar setup indicating that pyruvate oxidation is unaffected by the 5-AVAB exposure (Fig. 1b).

**Figure 2:**
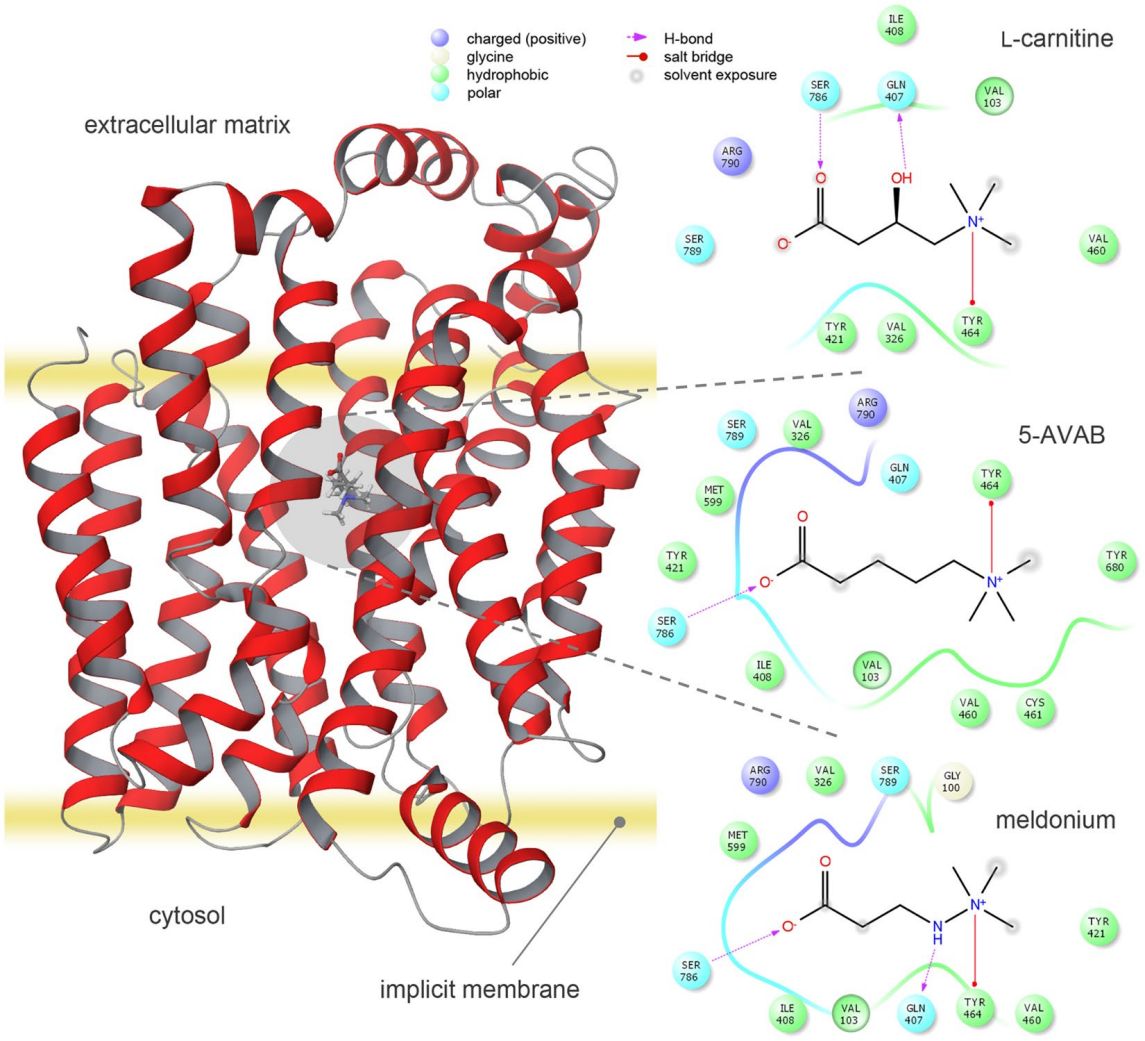
*In silico* docking of 5-AVAB to OCTN2. 5-AVAB docked into an *in silico* model of OCTN2 and predicted interactions between OCTN2 and 5-AVAB, L-carnitine and meldonium. TYR464, SER786 and GLN407 in the model correspond to TYR239, SER467 and GLN207 in the protein, respectively.

The molecular structure of 5-AVAB has similarity with meldonium (Fig. 2), which has been shown to decrease mitochondrial fatty acid β-oxidation and has been associated with improved cardiac mitochondrial function after ischemia^6^. The effect of 100 µM 5-AVAB on cellular oxygen consumption was similar to that of 100 µM meldonium (Fig. 1c). By contrast, 100 µM glycine betaine did not have significant effect on utilization of palmitate (Fig. 1c). Furthermore, when 2 mM L-carnitine was added to the cells, both 5-AVAB and meldonium induced reduction in β-oxidation disappeared (Fig. 1d), thus confirming that the effect is L-carnitine dependent. We also measured with mass spectrometry that 100 µM 5-AVAB significantly reduced cellular levels of L-carnitine (controls 61304 ± 8700 [mean ion abundance ± SEM], 5-AVAB treated 24264 ± 3062, p = 0.004, n = 5 in both groups) and acetylcarnitine (controls 39420 ± 1103, 5-AVAB treated 10620 ± 682, p < 0.001, n = 5 in both groups) in cultured mouse cardiomyocytes.

Finally, we performed a mitochondrial stress test in the presence of glucose and pyruvate to see if any of the betaine compounds had a direct effect on the function of the respiratory chain. None of the respiratory parameters were changed after the exposures indicating that 5-AVAB and meldonium influence specifically fatty acid β-oxidation in cardiomyocytes (Fig. 1e).

It should be noted, that even though OCR is an indirect measure of fatty acid oxidation, in the conditions where palmitate is the sole energy source in the Seahorse analysis, OCR is highly dependent of fatty acid oxidation, as shown by pharmacological manipulations done with etomoxir (Fig. 1a,c,d).

### 5-AVAB is a substrate for the membrane carnitine transporter

Potential mechanisms for the 5-AVAB induced impairment of fatty acid oxidation include inhibition of the carnitine transporter OCTN2 and inhibition of L-carnitine biosynthesis. However, a previous study has reported that 5-AVAB is unlikely to inhibit biosynthesis of L-carnitine from γ-butyrobetaine by γ-butyrobetaine hydroxylase^7^ while there was no previous research on 5-AVAB and OCTN2.

To provide insight into the possible mechanisms by which OCTN2 recognize their known substrates and 5-AVAB, we established *in silico* docking simulations in order to examine potential interactions. The template proteins, against which the *in silico* protein models were created, had a homology of 44% to OCTN2. The docking simulations for the validated model of OCTN2 showed an interaction between TYR239 and the trimethylamine group of 5-AVAB as well as between SER467 and the carboxylic acid group, similarly as suggested earlier for L-carnitine^9^ and meldonium in our model (Fig. 2). The predicted interactions between 5-AVAB and the OCTN2 suggest that 5-AVAB may be a potent inhibitor of carnitine uptake.

**Figure 3:**
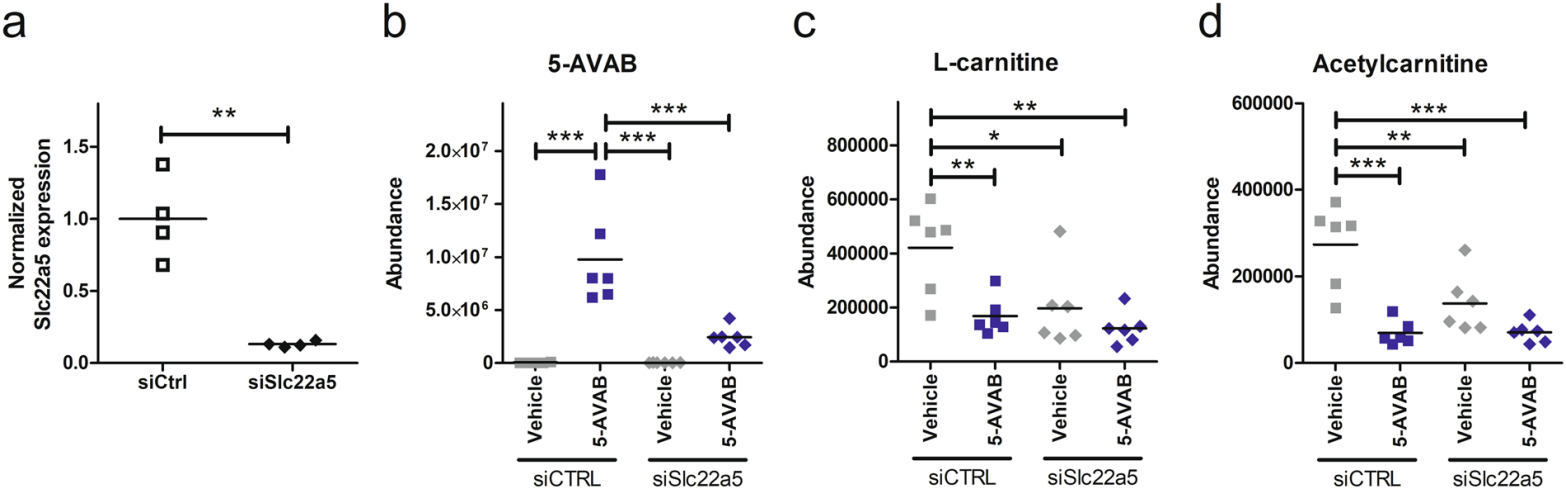
Effects of siRNA silencing of OCTN2 to intake of 5-AVAB. (**a**) Reduction of *Slc22a5* expression (gene for OCTN2) in siRNA transfected neonatal cardiomyocytes (normalized to expression in the siCtrl (nonsense) transfected cells). (**b**) Silencing of OCTN2 decreased cellular intake of 5-AVAB in cultured mice cardiomyocytes when compared to control siRNA treated cells. (**c**,**d**) Silencing of OCTN2 reduces the L-carnitine and acetylcarnitine levels in mice cardiomyocytes similar to treatment with 100 µM 5-AVAB. *p < 0.05, **p < 0.01; ***p < 0.001.

The *in silico* predicted high affinity of 5-AVAB to OCTN2 was further verified by silencing OCTN2 in cardiomyocytes with siRNA which resulted in similar reduction of L-carnitine and acetylcarnitine levels as the treatment with 5-AVAB (Fig. 3). Because OCTN2 silencing also reduced the cellular intake of 5-AVAB (Fig. 3b), it seems that 5-AVAB is a substrate for the OCTN2 and is blocking or slowing down L-carnitine intake, similar to meldonium^10^.

### 5-AVAB induced reduction of β-oxidation as a possible novel mechanism for beneficial functions of whole grains

5-AVAB induced identical inhibition of oxygen consumption due to β-oxidation of fatty acids and reduction of cellular L-carnitine levels in cardiomyocytes as structurally similar meldonium. It has been proposed that via this L-carnitine dependent mechanism meldonium forces cardiomyocytes to prefer glucose to fatty acids in energy metabolism, which protects heart in ischemic conditions^6^. Several other pharmacological agents which inhibit β-oxidation have been proposed for the clinical treatment of cardiac pathologies^11–13^. It should be noted that in the case of meldonium, which was also recently added to a list of prohibited drugs by world anti-doping agency, there is not a full scientific consensus on its efficacy as cardio protective or performance-enhancing agent^14^. One could also argue that reduction in β-oxidation is detrimental for the normal heart and skeletal muscle physiology since these tissues rely on fatty acid derived energy metabolism, especially during long lasting physical activity. These questions need to be addressed in the future studies determining the *in vivo* effects 5-AVAB in heart and skeletal muscles. It will also be necessary to gain understanding of its function in other tissues where L-carnitine has vital role such as in adipose tissue and kidney, which is mainly responsible for L-carnitine homeostasis in the body^15^.

The molecular mechanisms behind the association between whole-grain consumption and reduction of risk of Western lifestyle related diseases, like cardiovascular diseases, has been largely unknown^1–4^. Recently increased 5-AVAB levels in plasma and metabolically active tissues have been associated with diets rich in whole grains^5^. Here our main finding is that 5-AVAB, at physiological concentrations, reduces oxygen consumption caused by β-oxidation of fatty acids in cultured mouse cardiomyocytes, and that this effect is at least partly mediated by inhibition of OCTN2 by 5-AVAB. Moreover, 5-AVAB did not influence the utilization of glucose or pyruvate for mitochondrial respiration, indicating that 5-AVAB does not generally inhibit mitochondrial respiration. Whether the 5-AVAB present in heart and in other metabolically active tissues, or its induction by diets rich in whole grains, has significant effects *in vivo* on the energy metabolism at these sites requires further investigations. Nevertheless, we propose here an interesting and novel mechanism how diets rich in whole grains can participate in the regulation of cellular energy metabolism.

## Methods

### Synthesis of 5-AVAB and 5-AVAB-*d*9

*N*,*N*,*N*,-trimethyl-5-aminovaleric acid (5-AVAB) hydrochloride: 5-aminovaleric acid (0,85 mmol, 100 mg) and sodium hydroxide (2,95 mmol, 118 mg) were dissolved in 4 ml methanol-water 1:1 in a 5 ml microwave vial. Iodomethane (3,92 mmol, 244 µl) was added to the mixture. The vial was sealed and heated by microwaves at 60 °C for 60 min using a Biotage Initiator instrument equipped with an external IR-sensor to detect the reaction temperature. Solvents were evaporated and the solid was dissolved in 4 ml 1 M HCl and evaporated again to yield yellow solid, which was then washed several times with acetone to yield white solid. The product was dried in high vacuum (75 mg, 49%). ^1^H NMR (600 MHz, D_2_O) δ 3.28 (m, 2H), 3.04 (s, 9H), 2.41 (t, 2H), 1.76 (qt, 2H), 1.60 (qt, 2H); ^13^C NMR (125.8 MHz, CD_3_OD) δ 175.6, 67.9, 54.1 (br), 34.3, 23.8, 23.7.

*N*,*N*,*N*,-trimethyl-*d*_*9*_-5-aminovaleric acid (5-AVAB-d_9_): 5-aminovaleric acid (1.32 mmol, 155 mg) and sodium carbonate (6.62 mmol, 701 mg) were dissolved in 4 ml methanol-water 1:1. Iodomethane-d_3_ (5.39 mmol, 329 µl) was added and the mixture was stirred in a sealed vial for 48 h at room temperature. The sodium carbonate was filtered and the filtrate was evaporated. The white solid was treated with abs. ethanol and filtrated to remove sodium carbonate residues and the filtrate was acidified with 1 M HCl. After evaporation the solid was washed several times with Et_2_O to yield white solid (120 mg, 54%). ^1^H NMR (600 MHz, D_2_O) δ 3.19 (t, 2H), 2.34 (t, 2H), 1.69 (qt, 2H), 1.52 (qt, 2H); ^13^C NMR (125.8 MHz, D_2_O) δ 178.0, 65.75, 51.8 (br), 33.0, 21.7, 20.9.

### Mice and human tissue samples

C57BL/6J male mice were obtained from National Laboratory Animal Center (Kuopio, Finland). The animal experiments were approved by the Institutional Animal Care and Use Committee of the Provincial Government of Finland (license number 041003). Animal studies were performed following the guidelines of the Finnish Act on Animal Experimentation and Directive 2010/63/EU of the European Parliament. At 28 weeks of age the mice (n = 5) were fasted for 8 ± 0.5 hours and sacrificed by decapitation after made unconscious by CO2 gas. The heart tissues were rinsed with physiological saline, wrapped in aluminum foil, and snap frozen in liquid nitrogen and kept at −80 °C.

The collection of the human heart samples was approved by the Health and Scientific Research Ethical Committee of Budapest and conducted in accordance with the guidelines of the Declaration of Helsinki. Atrial and ventricular tissue samples were obtained from non-diseased donor hearts unsuitable for transplant. Two atrial and ventricular samples were from same donor hearts and one ventricular sample from a third donor heart. Samples were snap frozen in liquid nitrogen and stored at −80 °C.

### Tissue sample preparation for MS-analysis

Frozen tissue samples were cryo-ground in 2 ml microcentrifuge tubes with 4 mm stainless steel beads in a precooled 2 × 24 adapters that was shaken for 45 s at 30 Hz using TissueLyser II (Qiagen Finland, Helsinki, Finland). Samples containing 100 mg (±2 mg) of tissue powder were cryo-weighted into 1.5 mL microcentrifuge tubes and 80% methanol was added (v/v H2O, LC-MS Ultra CHROMASOLV®, Fluka) in a ratio of 300 µL solvent/100 mg tissue. The samples were shaken for 20 min and centrifuged for 10 min at 4 °C (13 000 rpm) and supernatants were filtered using 0.2-µm Acrodisc® Syringe Filters with a PTFE membrane (PALL Corporation) and stored at −20 °C until LC-MS analyses. Standard and internal standard (5-AVAB-d_9_) were solubilized and diluted in 50% ACN to get stock solutions and intermediate dilutions (stored in −20 C°). Standard intermediate dilutions were diluted further 1:25 in 80% MeOH and internal standard was diluted 1:2500 in ACN to get working solutions. For analyses, 8 µL of standard working solution or sample was mixed with 32 µL ice-cold internal standard working solution.

### Targeted mass spectrometry method for measurement of 5-AVAB

The HPLC system (Agilent 1200 Series Rapid Resolution LC System) consisted of a solvent degasser, a binary pump, a thermostated column compartment and an autosampler. Autosampler temperature was fixed at 4 °C. The chromatographic separation was performed with the AcQuity UPLC BEH Amide 1.7, 2.1 × 100 mm (Waters). The column temperature was set 45 °C. As mobile phases 50% ACN in H_2_O including 20 mM ammonium formate and 0.25% formic acid (A) and 90% ACN in H_2_O including 20 mM ammonium formate and 0.25% formic acid (B) were used. The initial gradient condition of the elution was 100% B for 2.5 min, which was decreased linearly to 0% in 7.5 min, increased to 100% in 0.01 min and kept constant for 2.49 min until next run. In the run 1 µl of sample/standard was injected and the chromatographic separation was performed with flow-rate of 0.6 ml/min. For mass analyses Agilent 6410 Triple Quad LC/MS with an electrospray source (Agilent Technologies) was used. The drying gas (nitrogen) temperature was set 300 °C, gas flow 8 L/min and capillary voltage 4000 V. The collision gas was argon. The quantitation was made using positive ion mode and multiple reaction monitoring (MRM) with MassHunter Workstation Acquisition software (Agilent Technologies). The ion transitions observed were for 5-AVAB 160.1- > 101.0 and for 5-AVAB-d_9_ 169.2->101.0 with collision energy of 20 V.

### Neonatal mice cardiomyocyte isolation and culture

One to two days old mice from C57BL/6JOlaHsd strain were sacrificed by decapitation, ventricular tissue was cut into small pieces and dissociated into single cells with enzyme solution containing 2 mg/mL collagenase type II (Worthington, NJ, USA) and 2 mg/mL pancreatin (Sigma-Aldrich, MO, USA) in 37 °C for 90 min. Dissociated cells were plated on dishes coated with solution containing 12.5 µg/mL fibronectin (Sigma-Aldrich, MO, USA) and 200 µg/mL gelatin (Sigma-Aldrich, MO, USA) in cardiomyocyte culture medium (CCM, Dulbecco’s Modified Eagle Medium (Thermo Fisher Scientific, MA, USA), 10% fetal bovine serum (GE Healthcare Life Sciences, UT, USA), 100 U/mL penicillin-100 µg/mL streptomycin (Thermo Fisher Scientific, MA, USA)). Cells were cultured for at least 24 hours after the isolation before exposure to the studied compounds. All exposures were done in the cardiomyocyte culture medium.

Gene silencing by siRNA was performed with Lipofectamine® RNAiMAX Transfection Reagent (Thermo Fisher Scientific, MA, USA) according to manufacturer’s instructions using commercially available siRNA oligos (Qiagen, Hilden, Germany; Slc22a5, cat. no. SI01420762; siCtrl, cat. no. 1027280). For siRNA studies, statistical significance was evaluated with one-way ANOVA followed with Tukey’s post-hoc test or with Student’s t-test when evaluating only two groups (α level 0.05).

### Analysis of cell respiration

Oxygen consumption in cultured neonatal cardiomyocytes was measured with Seahorse XF24 Analyzer (Agilent Technologies, CA, USA). After exposure to the betaine compounds, cells cultured on XF24 Cell Culture Microplates (Agilent Technologies, CA, USA) were washed once with XF Base Medium (Agilent Technologies, CA, USA) (pH 7.4) and 0.5 mL of assay medium (XF Base Medium supplemented with energy substrates) was added to the wells. Experimental groups were supplemented with the respective betaine compound also during the assay. In the experiments determining the utilization of fatty acids, assay medium was supplemented with 2 mM GlutaMAX (Thermo Fisher Scientific, MA, USA) and 200 µM palmitic acid (Sigma-Aldrich, MO, USA) conjugated to bovine serum albumin (BSA, Sigma-Aldrich, MO, USA). Method for conjugation of palmitate to BSA was acquired from publication by Belke *et al*.^16^. In the mitochondrial stress test assay medium was supplemented with 2 mM GlutaMAX, 4.5 g/L glucose and 0.11 g/L sodium pyruvate (Sigma-Aldrich, MO, USA). Compounds injected to the cells during the analysis of respiration (final concentration, all purchased from Sigma-Aldrich): 40 µM etomoxir, 1 µM oligomycin A, 1.5 µM Carbonyl cyanide 4-(trifluoromethoxy)phenylhydrazone (FCCP), 5 µM antimycin A. In the mitochondrial stress test, respiration originating from ATP production was calculated as a difference between basal and oligomycin A induced OCR, proton leak as a difference between oligomycin A and antimycin A induced OCR, and mitochondrial reserve as a difference between FCCP induced and basal OCR. Statistical significance was evaluated with one-way ANOVA followed with Tukey’s post-hoc test (α level 0.05).

### Mass spectrometry of cell samples

After 24 h exposure to 100 µM 5-AVAB, neonatal mouse cardiomyocytes were washed once with Dulbecco’s phosphate buffered saline (PBS, cat.no. D8537, Sigma-Aldrich, MO, USA). The cells were scraped from the dishes in ice cold PBS, centrifuged and stored in −70 °C as pelleted cell material until analysis.

The cell samples were analyzed by the semi-targeted ultra-high performance liquid chromatography quadrupole time-of-flight mass spectrometry UHPLC-qTOF-MS system (Agilent Technologies, a 1290 LC system, a Jetstream electrospray ionization (ESI) source, and a 6540 UHD accurate-mass qTOF spectrometer). We used hydrophilic interaction chromatography (HILIC) and positive ionization. The sample tray was kept at 4 °C during the analysis. The data acquisition software was the MassHunter Acquisition B.04.00 (Agilent Technologies). The mass spectrometry method have been described in detail earlier^17^. Data processing was carried out using Profinder (Agilent B.08.00), which enabled manually picking up mass spectrometry peaks associated with 5-AVAB, L-carnitine and acetylcarnitine. Compounds were identified by comparison of retention times and use of targeted MSMS spectra, which were compared to spectra of commercial and synthetized chemical standards: 5-AVAB: RT 2.2 min and MSMS-spectra (20 V) 55.0554 (100%, relative abundance), 60.0822 (58%), 101.0610 (55%), 83.0503 (32%), 59.0500 (27%), 160.1348 (15%); L-carnitine: RT 4.6 and MSMS-spectra (20 V) 60.0812 (100%), 103.0393 (91%), 43.0183 (72%), 85.0289 (70%), 162.1124 (46%), 102.0919 (44%), 57.0339 (21%); and acetylcarnitine: RT 2.7 and MSMS-spectra (20 V) 85.0290 (100%), 60.0818 (11%), 204.1228 (5%), 145.0497 (5%). Statistical significance was evaluated with one-way ANOVA followed with Tukey’s post-hoc test or with Student’s t-test when comparing only two groups (α level 0.05).

### Molecular modelling simulations

Comparative modelling of OCTN2: PSI-BLAST^18^ was used to search for homologous template proteins of OCTN2 (organic cation transporter 2, OCTN2; solute carrier family 22 member 5, SLC22A5). The alignment of the target sequences to the selected template proteins was performed in Discovery Studio 4.5 Client (Dassault Systèmes BIOVIA, San Diego, USA). The best alignment was chosen based on visual inspection and the knowledge of conserved regions in the sequence. Subsequently, the model of the target protein was constructed with the MODELER 9.14. module (https://salilab.org/modeller/) implemented in Discovery Studio, using the template protein with the highest homology according to PSI-BLAST. Initially, 100 models were created with high optimization level, and the model with the lowest PDF total energy was chosen for further refinement and evaluation. Loop refinement was performed using the Looper algorithm in Discovery Studio with a maximum of 20 models created for each refinement run. For comparison, a second model of each protein was created with the I-TASSER online server^19,20^ using default restraints and templates. The two models were assessed and compared in Discovery Studio using the DOPE score and the Ramachandran plot^21,22^. The best model was then selected to be used in the docking simulations.

Docking of 5-AVAB and structural analogues: QM-polarized ligand docking (QPLD) was performed in Maestro 10.6 (Schrödinger LLC, New York, USA) to study the recognition of 5-AVAB, D-carnitine, L-carnitine (known substrate), 5-dimethylaminovaleric acid, glycine betaine, meldonium and choline by OCTN2. Prior to docking, the grid box was centered to residues most likely responsible for ligand recognition based on the mutational data from literature^23^ and sitemap analysis. For the Glide docking, the initial charges were created with a semi-empirical method at standard precision. Prior to redocking, the quantum mechanical polarized charges were generated with the Jaguar module of Maestro using fast settings. The final selection of poses was carried out by using Coulomb–van der Waals interactions.

## Data Availability

The datasets generated and analysed during the current study are available from the corresponding author on reasonable request.
